# The use of open source GIS algorithms, big geographic data, and cluster computing techniques to compile a geospatial database that can be used to evaluate upstream bathing and sanitation behaviours on downstream health outcomes in Indonesia, 2000–2008

**DOI:** 10.1186/s12942-018-0164-6

**Published:** 2018-12-14

**Authors:** Stuart E. Hamilton, John Talbot, Carl Flint

**Affiliations:** Department of Geography and Geosciences, Salisbury University, Salisbury, MD 21801 USA

## Abstract

**Background:**

Waterborne diseases are one of the leading causes of mortality in developing countries, and diarrhea alone is responsible for over 1.5 million deaths annually. Such waterborne illnesses most often affect those in impoverished rural communities who rely on rivers for their supply of drinking water. Deaths are most common among infants and the elderly. Without knowledge of which communities are upstream of a community, upstream sanitary and bathing behaviors can never be directly linked to downstream health outcomes including disease outbreaks. Although current GIS technologies can answer the upstream question for a limited number of downstream communities, no systematic way existed of labeling each downstream village with all its upstream contributing villages along river networks or within basins at the large national scale, such as in Indonesia. This limitation prohibits macro analyses of waterborne illness across developing world communities globally.

**Results:**

This novel method approach combines parallel computing, big data, community data, and open source GIS to create a database of upstream communities for 50,000–70,0000 villages in Indonesia across four differing periods. The resultant village database provides information that can be tied to the Indonesian PODES health and behavior surveys in each village to connect upstream sanitary behaviors to downstream health outcomes. We find that the approximately 250,000 communities analyzed across the four periods in Indonesia have a combined total of 13.7 million upstream villages. The average number of upstream villages per village was almost 55, the maximum number of upstream villages for any single village was over 5300.

**Conclusions:**

Advances in big-data availability, particularly high-resolution elevation data, the lowering of the cost of parallel computing options, mass survey data, and open source GIS algorithms that can utilize parallel processing and big-data, open new opportunities for the study of human health at micro granularities but across entire nations. The database generated has already been used by health researchers to compute the influence of upstream behaviors on downstream diarrhea outbreaks and to monitor avoidance behaviors to upstream water behaviors across all downstream 250,000 Indonesian villages over 4 years, and further waterborne health analyses are underway.

## Background

Waterborne deaths remain one of the primary causes of mortality and illness in the developing world [1, 2] with diarrhea accounting for over 1.5 million deaths annually [3]. Such water-borne disease tends to affect the young in the poorest regions of the developing world. Upstream behavior such as water bathing, water sanitation, and waste disposal affects the health of downstream populations in areas where drinking river water or bathing in river water is commonplace. To account for upstream behavior in health analysis, researchers and practitioners require databases that can account for actions of each upstream community for each community of observation. That is, what villages or communities are upstream of my community? Traditional GIS methods can answer this question for a limited number of communities, but in the cases of Indonesia, the task requires new GIS approaches and access to high-performance computing. For example, over 70,000 individual villages existed in Indonesia in 2008. The question of what villages are upstream of every other village is the challenge addressed in this paper. For researchers and practitioners to fully account for upstream behaviors in downstream health outcomes, particularly as they pertain to waterborne disease, this question needs answering.

Although reliable human health survey exists for all Indonesian villages, a comprehensive upstream database for every village is lacking. In rural areas of the developing world, precise networks of existing river structures, particularly minor rivers that are more likely to be used for water consumption, are just not available [4]. Even when reliable river networks do exist, they exist in isolation to the village locations and associated health data. Yet, it is household pollution from residents that are likely the primary source of river pollution, and hence adverse health outcomes, across Indonesia. For example, approximately 66% of the Citacum River’s BOD is estimated to come from household waste [5], far outstripping both agricultural and industrial inputs. In addition to the disposal of household waste, river bathing is likely a source of upstream contaminants leading to downstream health issues. Within the Ganges River system it has been shown that upstream river bathing likely results in increases in coliform bacteria which are linked to downstream incidents of nausea, vomiting, diarrhea [6, 7]; with infants and those with compromised immune systems. Additionally, river bathing of infants after soiling is frequent and problematic to downstream communities as well as river bathing. It worth to mention that the use of rivers for household waste disposal remaining almost entirely unregulated in Indonesia [8].

### Study area

The study area is all of Indonesia. The communities or villages are defined as polygonal areas by the Indonesian Central Bureau of Statistics Village Potential Statistics (PODES) enumeration surveys of 2000, 2003, 2006, and 2008 and known as Desas. To allow for the realistic generation of modeled water flow, all countries that share an island with Indonesia are included in the hydrological analysis but not in the village-level analysis. These countries are all or parts of Brunei, Papua New Guinea, Timor-Leste, and Malaysia. An additional geographic constraint was inclusion in the Shuttle Radar Topography Mission (SRTM) 1 arc-s global void-filled elevation dataset, that likely omits some small islands that may be inhabited but may not contain many upstream communities due to the small size of the islands. The number of villages enumerated varies from a low of 56,579 in 2003 to a high of 71,282 in 2008. In 2008, this equated to 1,654,043 km^2^ of Indonesia’s 1,904,569 km^2^ land area, or 87%, of Indonesia being designated as within an analyzed village. The total population analyzed in 2008 is 229,171,551; this is likely close to 100% of the population of Indonesia in 2008. The national census in 2010 reports 237,641,334 million people and an annual growth rate during the 2000s of 1.54%.

## Methods

The methodology designed focused on obtaining reliable upstream village information for each of the 56,579–71,282 villages present in Indonesia across the four differing years of analysis. The calculation of all upstream villages for each village required the construction of a digital terrain model (DTM) with hydrologic enforcement and then utilizing the resultant DTM to delineate the upstream area for each village. The hydrographic model of Indonesia had to be constructed as part of this project as no systematic, seamless, high-quality and international hydrography dataset exists [9] at the required resolution. Once the upstream area for each village is delineated, it is relatively straightforward to extract all communities that are in this upstream area.

### Elevation

The core elevation layer for the Indonesian hydrologic model created is the recently released SRTM 1 arc-s global void-filled elevation dataset [10]. These elevation data are derived from the Space Shuttle mission of 2000 but were only released at a 1-arc-s resolution for Southeast Asia in 2015. These elevation data were obtained in 10° raster tiles, with a pixel size of approximately 30 m, and in an unprojected coordinate system that is based on latitude and longitude. The first step was to combine the individual tiles into a single seamless digital elevation model (DEM) that contained all the elevation measures for Indonesia and for any countries whose islands are shared with Indonesia. These additional countries are required to create a representative hydrologic model of Indonesia as if omitted the hydrologic model may not be able to determine flow directions correctly resulting in an unreliable representation of upstream villages within Indonesia.

Once a singular DEM for the region was created, the DEM was reprojected into a suitable equatorial Mollweide coordinate system, based on meters, for all of Indonesia and surrounding countries. Ninety-meters was selected as the most suitable horizontal analysis resolution after testing a sample of the approximately 30 m input data at the raw 30 m resolution as well as at 60 m, 90 m, and 120 m aggregates of the data. The 90 m selection was a compromise achieved by comparing the output flow lines generated from each differing resolution for the sample area against a river layer provided by the World Resources Institute (WRI) that was constructed from the manual digitization of aerial photography. The desired resolution had to match the WRI river locations accurately, be substantially smaller than the smallest village in the PODES, and allow for computation of upstream areas within a reasonable timeframe using the cluster computing resources available. The final analysis DEM at 90 m resolution contains 3.47138 × 10^8^ terrestrial pixels. This value is the sum of elevation pixels, and hence derived drainage pixels, that exist over the totality of Indonesia. This DEM value is the number of pixels that will have to be queried, at multiple different times, to create accurate watersheds for each village in the database. This compromise resolution has a large enough cell-size to process more rapidly but is resolute enough to allow for accurate depiction of drainage at the village scale, as well as containing enough detail for even the smallest villages to contain multiple pixels.

Although the SRTM product is labeled as void-free, isolated voids do still exist in the Global SRTM 1 arc-s product [11]. We filled these voids using the r.nulls algorithm, this algorithm passes a regularized spline through the neighbors of the null pixel and then estimates the omitted null value from the interpolated neighborhood values [12]. Once void filling is complete, the output is a 90 m resolution, gap-filled, seamless, and projected DEM for all of Indonesia and all islands of which Indonesia is a part.

### Terrain and hydrology

**Fig. 1:**
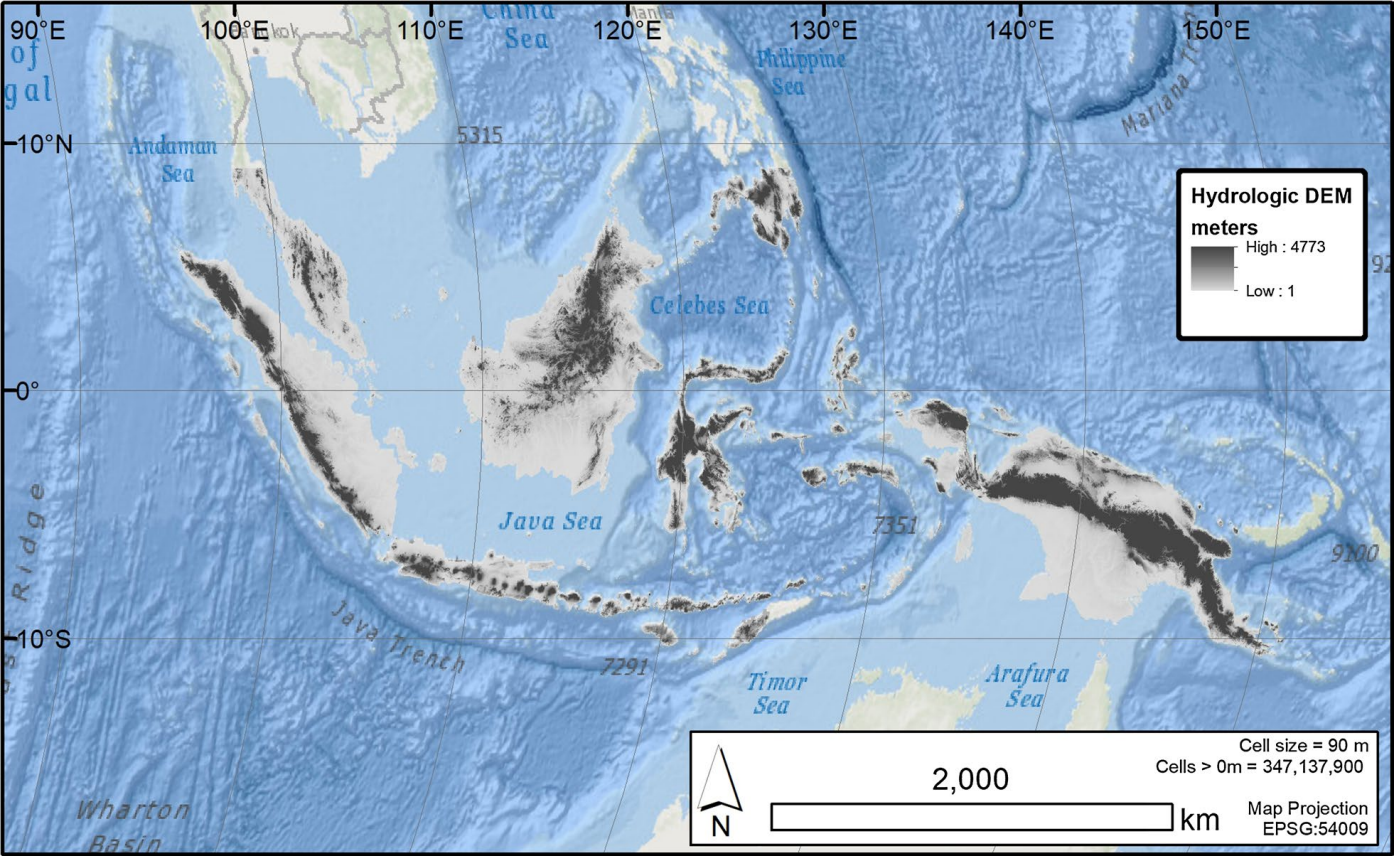
The final DEM used to delineate upstream villages for each of the villages in Indonesia. The DEM contains over 347 million measures of elevation and is hydrologically enforced

Once void-filling was complete, hydrologic enforcement was completed using the methods outlined by Tarboton et al. [13]. This process allows for modeled flow to be preserved in areas of real or spurious topographic depressions. Figure 1 represents the void-filled, hydrologically enforced, seamless 90 m resolution DEM for all the islands of which Indonesia is a part. Once hydrologic enforcement was complete, we used the D8 algorithm [14], within the open-source r.watershed process, to assign flow directions across the dataset and obtain the number of pixels that flow through every other pixel across the entire DEM. These outputs are commonly referred to as the flow direction layer and the flow accumulation layer respectively. The r.watershed algorithm was selected as it has proven effective in similar studies in other countries with high-relief topography when used to depict river channels and flow accumulation from SRTM data with canopy interference likely to be present [15]. Additionally, the r.watershed algorithm is open-source and allows us to customize the algorithm for use in a parallel processing environment.

**Fig. 2:**
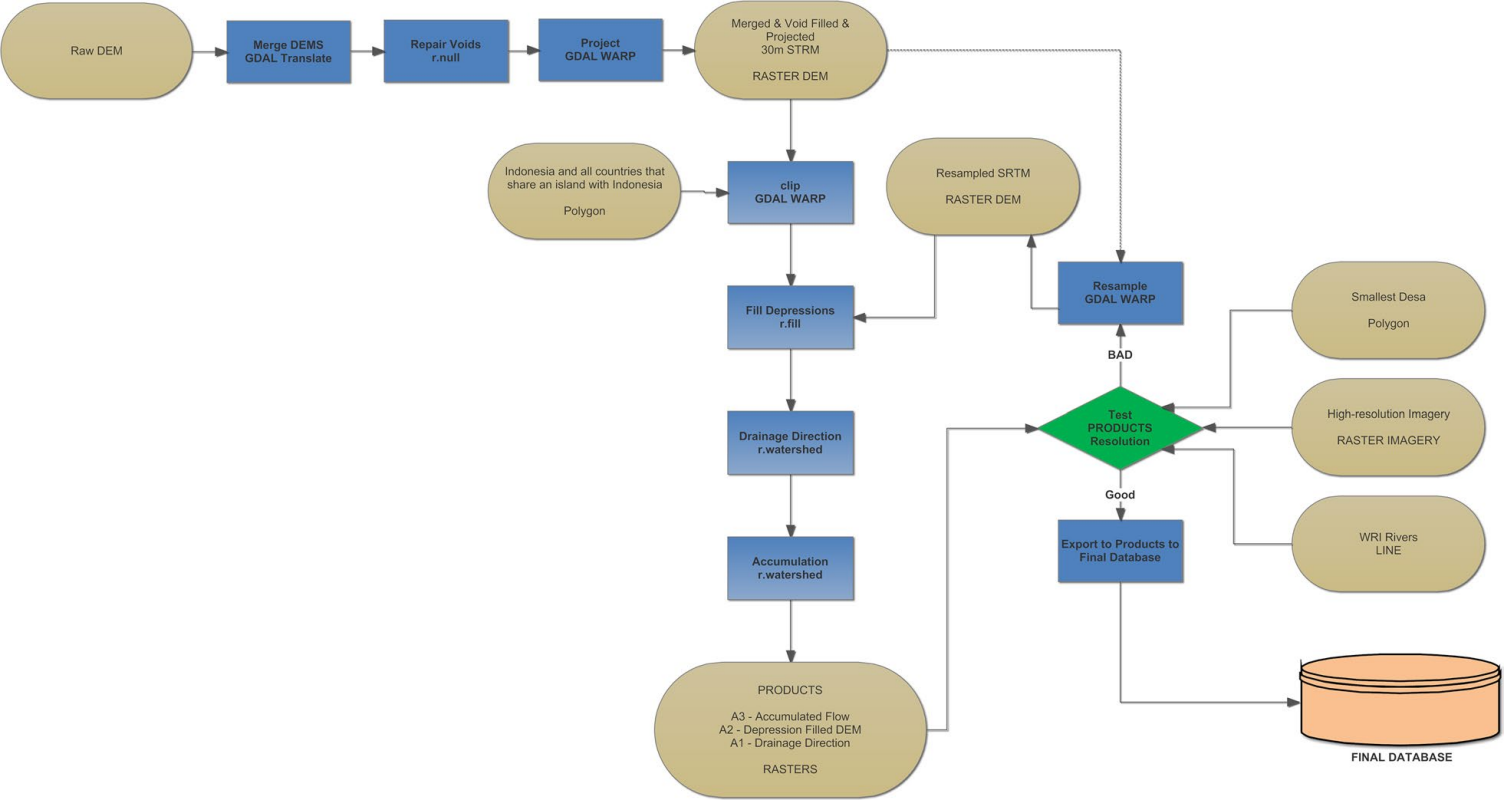
The DEM and hydrology process flow chart

**Fig. 3:**
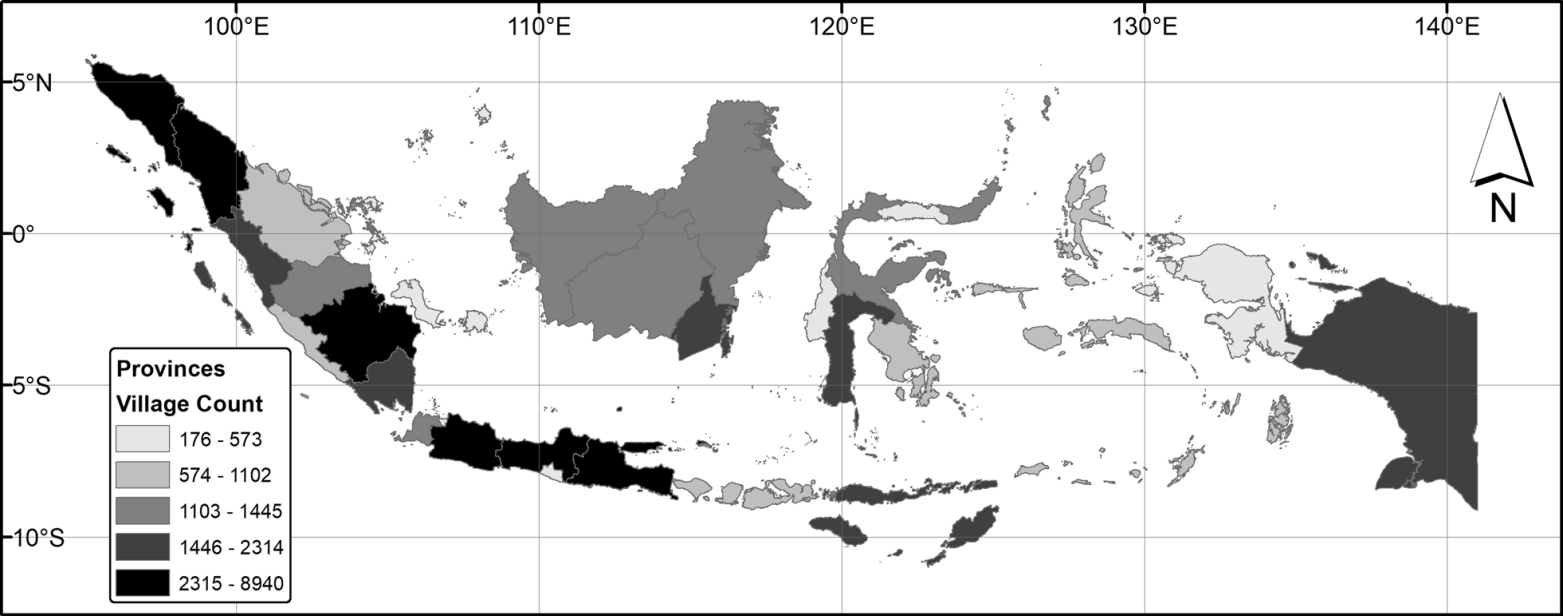
The distribution of villages used in this analysis. The village data is from 2006 and aggregated to the province level

Figure 2 summarizes the process from obtaining the DEM to the final output hydrology products. The Fig. 3 flowchart depicts the input data, the processes, the FOSS algorithms implemented, the decision points, and the output datasets. The full resolution model is available in the Harvard Dataverse repository.

### Village data

The PODES dataset provides information about village characteristics for all of Indonesia [16], and since 1980, is managed by The Indonesian Central Bureau of Statistics. PODES collection complements the population census, the economic census, and the agricultural census following similar collection procedures. Since 2003, village-level data was collected in its entirety. The PODES questionnaire consists of two components. They are the core data, which is obtained in every census, and the module data that is collected only in the implementation of agriculture census.

PODES data was provided in polygon shapefile format by The Indonesian Central Bureau of Statistics. The dataset consists of one shapefile for each of 2000, 2003, 2006, and 2008. Each village is a row in the shapefile attribute table. Preprocessing of the village polygons included completing logical topological fixes such as eliminating spurious gaps between the villages, correcting overlapping village boundaries, and removing duplicate village polygons. To increase computational processing efficiency, all attribute data was transferred away from the village polygons at this stage aside from each village’s unique identifier. Once this process was complete, the village polygon data were then reprojected from their unprojected coordinate system into the Mollweide projection utilized in the SRTM-derived DEM. The final village counts, mapped to the provincial level, are depicted in Fig. 3.

### Upstream village delineation

Big-data is generally used to refer to datasets that contain so many records that they are too large for traditional data analysis hardware, software, and tools to provide insights. Large geographic datasets have the increased complexity that they are often too large to be opened using high-performance desktop computers. It is the delineation if upstream villages, for each downstream village, that requires big-data approaches as traditional approaches cannot process such data promptly. It is not the complexity of the delineation of upstream villages that is the issue, it is the sheer number of computations required, and the data storage requirements that make it problematic.

The number of upstream villages determination likely requires both big-data, and cluster computing approaches. To tabulate the number of upstream geographies that exist for each downstream village is both an unknown and potentially in the many billions range. The maximum theoretical number of upstream villages would be achieved if all individual villages were on a single long river, with no river branches, running downhill with the villages in a sequence one after the other. In this scenario, the village with the highest elevation would have no upstream villages. The next village down the river would have one upstream village associated with it; the net village downstream would have two upstream villages associated with it, the next village three upstream villages, and so on. Such a relationship can be written as a simple arithmetic sequence (Eq. 1). Using Eq. 1, we can calculate the theoretical maximum number of upstream villages. The theoretical maximum number of upstream villages is 1,647,695,715 for 2000, 1,600,619,910 for 2003, 2,151,319,215 for 2006, and 254,0597,403 for 2008. As each year is treated as a separate event, the theoretical number of potential upstream village boundaries to be extracted, for each downstream village across the entire dataset is 6,294,184,223.

1 $$a_{n} = a_{1} + {\text{f}}\left( {{\text{n}} - 1} \right).$$

The minimum theoretical number of upstream villages is merely the sum of the villages. In this scenario, imagine each village sitting on a small river with no upstream villages, it may help to envision the villages evenly spaced along the shoreline of a circular island that increase in elevation towards the center of the islands, such as a volcanic island. In this hypothetical scenario, no villages would have upstream neighbors. Therefore, before processing it can be assumed the number of upstream villages falls between 250,860 and 6,294,184,223. Each of these villages has a distinct polygon boundary, numerous attributes, and will require an upstream tale for all upstream villages to be constructed. This number of geographies, relations, and attributes fall squarely in big geodata analysis.

**Fig. 4:**
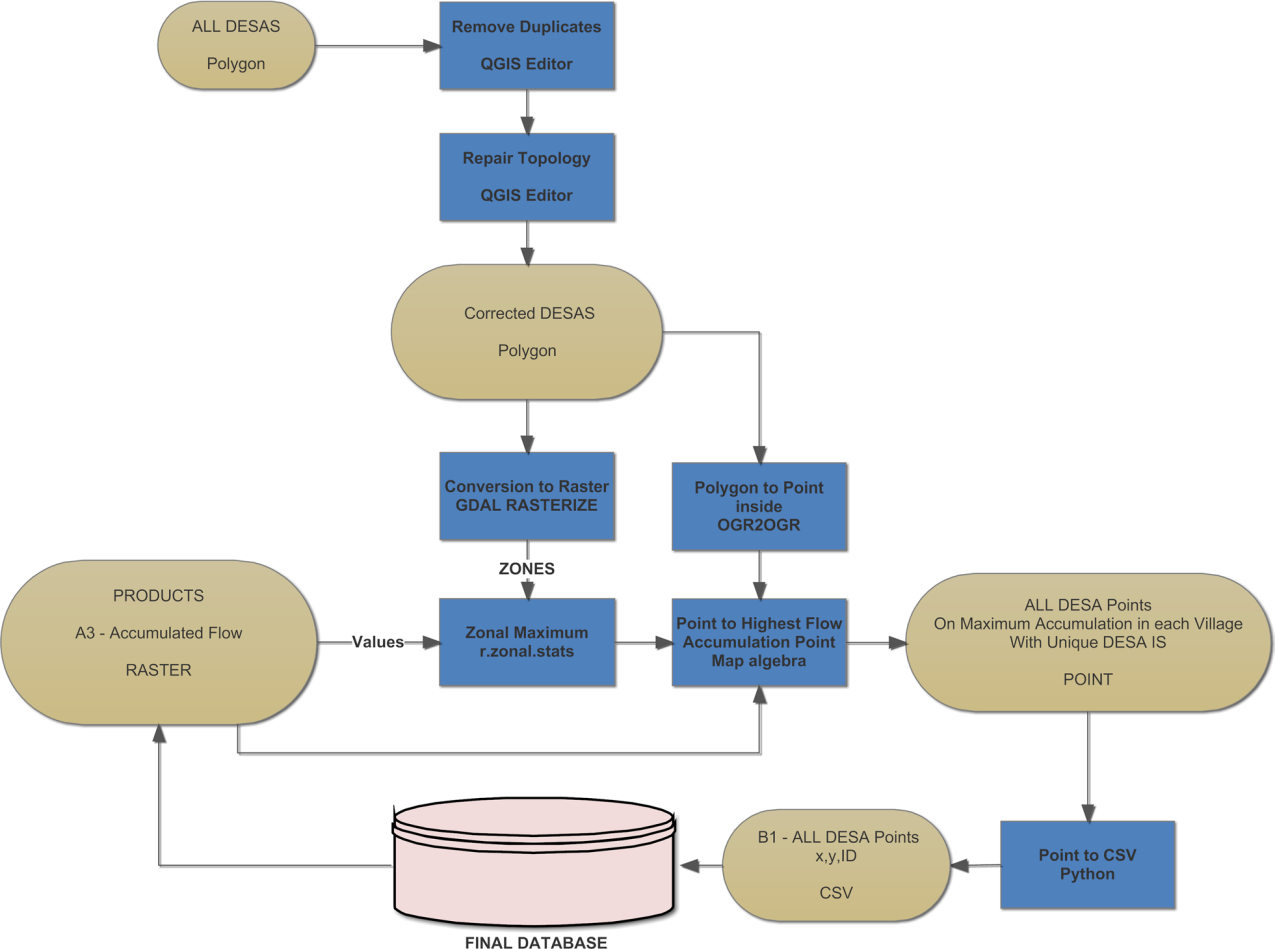
The hydrology and input village data flow chart

The delineation of upstream areas for each village requires an origin point within each respective village from which to delineate its upstream area. Within hydrologic analyses, such locations are most often referred to as pour points. To create pour points, the village polygons were converted into points, and the points were then located on the highest flow accumulation pixel within each village boundary. This was achieved using a simple zonal maximum function combined with a conditional statement using simple QGIS map algebra. That is, the village pour points were located on the highest possible flow location within that village polygon. Placing the village points on the highest possible accumulation site allows for the future upstream areas generated to capture all potential upstream regions. For example, if two major rivers come together in a village then using a pour point location in any other position within a village than the highest flow accumulation has the potential to result in large upstream areas being omitted from the final upstream delineation for that village. Finally, the point files were deconstructed into a simple table with each village assigned a unique coordinate pair based on their maximum potential flow pixel retaining their unique identifier. This simple three-column village table, containing all 71,000 village coordinate pairs and unique identifiers, provided the input to the hydrologic analyses. The combining of the village data with the hydrological data is modeled in Fig. 4. Figure 3, output A3, carries across to this model as the primary input. Figure 4 flowchart depicts the input data, the processes, the FOSS algorithms implemented, the decision points, and the output datasets. The full resolution model is available in the Harvard Dataverse repository.

From this point forward, all operations are conducted in the cluster environment. Once an Indonesia-wide flow direction, Indonesia-wide flow accumulation, and each village pour point had been created, we used the r.water.outlet algorithm in GRASS in conjunction with the flow direction output from the D8 algorithm to delineate the entire portion of Indonesia where water could originate that potentially flowed into each village via overland flow. The D8 process resulted in an upstream area polygon for that particular village. From the village’s upstream area, we then extracted all other villages that were wholly or partially contained within the upstream region of the village being analyzed. Once extracted, the upstream village unique ID was stored in a simple table structure with a one-to-many relationship with each village potentially having many villages upstream. This process was conducted iteratively for all 57,405 villages defined in the 2000 data, and next for all 56,579 villages in the 2003 data, then for all 65,594 in the 2006 data, and finally for all 71,282 villages in the 2008 data. The process had to be repeated for all years due to village redistricting between years. The villages are most numerous around the islands of Java, North and South Sumatra, and Southern Sulawesi with fewer villages on Papua, Eastern Kalimantan, and other remote islands.

### Spatial processing

The process of calculating the upstream area for delineated each village involved a version of the GIS operation most often referred to as a watershed or basin analysis [17]. Watershed delineation, involves an iterative search of the flow direction layer, beginning at the pour point and iterating away from this region until all flow can be assigned as away or towards the pour point. The combined area that flows into the pour point is defined as the upstream area. As our pour points are located on the highest modeled flow passing through each village, a sizable upstream area is likely returned for all villages with flow, and those on the coast will return upstream areas covering vast tracts of land on the island scale.

Testing on the dataset revealed that it would take more than 10 min to delineate the upstream area of a coastal village with a large upstream area and the average time was found to be slightly less than two minutes per village across all villages. This test was conducted on 100 randomly selected villages using commercial off-the-shelf ArcGIS 10.3.1 and ArcGIS Pro software on a 64-bit quad-core high-performance desktop computer with 64 GB of available memory in a 64-bit processing environment. This time factor is not surprising, as, at 90 m resolution, the flow direction raster that required searching contains 3.47138 × 10^8^ terrestrial pixels, as described above. Although many of these pixels are located on other islands or outside of the village basin and could be excluded this still leaves 1.38855 × 10^9^ pixels to be analyzed across all 4-years to ascertain the upstream areas. This value is merely the number of elevation pixels, and hence drainage pixels, present multiplied by the number of years within the analysis.

The test indicated it would require almost 1 year of processing time to process the 250,860 villages’ (which is the sum of all villages across the four analysis years) unique upstream areas, although this could theoretically be reduced by approximately to 120-days by preserving the upstream output area for each village in 2010 and using this for prior years, this though could lead to errors due to redistricting. Even if preservation of upstream villages from year-to-year was attempted, it did not provide the substantial time saving required, as 120-days per processing run remained unsatisfactory. Additionally, storing each polygonal watershed would require writing each of the village upstream areas to physical media. This process was found to substantially increased the time per delineation and still resulted in processing time between 6 and 8 months, requiring many thousands of large hard drives.

To overcome this issue, we used a parallel processing solution across 64 physical cores, and 64 hyper-threaded simulated cores in a wholly 64-bit environment allotting 8 GB of ram per village. Each output was temporarily stored in RAM with only the final upstream table being written to physical media. Use of this environment reduced the processing time from approximately 1-year in off-the-shelf GIS packages to just under 48-h using open-source solutions in an embarrassingly parallel environment.

GRASS 7 was utilized as the GIS engine within a Linux environment. GRASS operations were managed using bash and input data distribution was handled with a Python script. All processes were run in parallel using GNU parallel management software. Within GRASS 7, r.water.outlet was used as the upstream algorithm and r.watershed as the hydrologic enforcement, flow accumulation, and flow direction algorithm. Once the upstream GIS analysis was complete the data for the villages was reconnected to the village census data using the primary and foreign keys for analysis in STATA. C++ was used to calculate distances and STATA to assign macro-basin identifies. ESRI ArcGIS 10.3, in a windows environment, was used for data validation tasks. The compiled DEM, the tabular output data, and all code are available for download at https://dataverse.harvard.edu/dataverse/Indonesian_Watersheds and are available for use under CC BY-SA license.

**Fig. 5:**
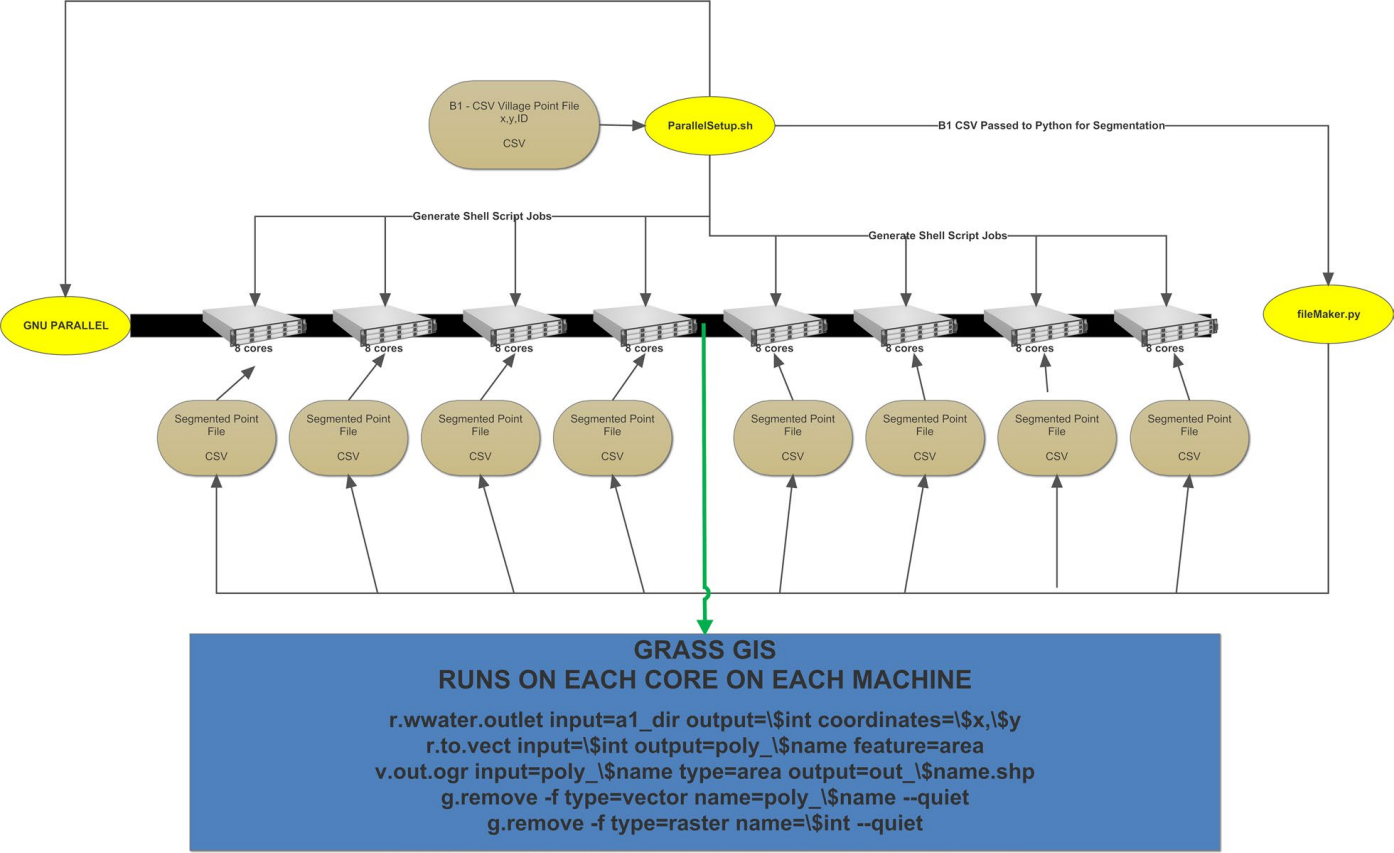
The cluster computing and parallel processing analysis flow chart

Figure 5 represents the cluster computing analysis that is at the core of this methodology. Figure 4, item B1 is passed to each of the computers using python, Fig. 3, A1, A2, and A3; the hydrology output data, from Fig. 3 reside on each machine before processing. All Grass processes are run in RAM, and no data is retained from each process once it is created aside from the final CSV that is a simple listing of the input Desa and then all upstream Desas delimited by a comma. The 64-individual output CSVs are merged into 8 CSVs at the computer level before being moved to one machine and merged into a single output CSV for all Desa. Figure 5, is iterated four times, one four each analysis year. Hence, the final result is 4 CSV files, each of which is made available in the Dataverse.

## Results

This manuscript only presents the results of the method; it does not discuss the results of the health outcomes.

The complete analysis process was reduced in time from approximately 1 year via traditional methods, to 2-days using FOSS algorithms, cluster computing, and a variety of different coding environments. Each Desa was coded with its entire upstream Desa list which allows for analysis of health outcomes with appropriate Desa-level ancillary survey data. The model can be applied to any location where regions are defined by a polygonal boundary, for example, US blocks, block groups, zip codes, tracts, or cities.

**Fig. 6:**
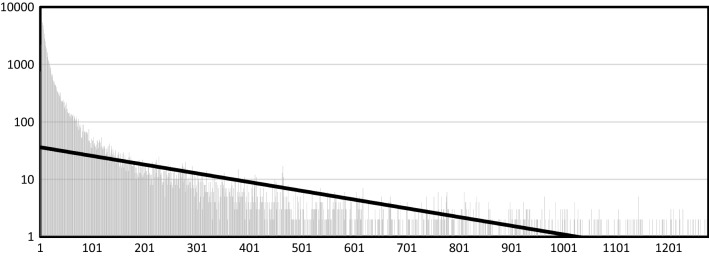
Upstream frequency log_10_(x). The x-axis represents the number of villages. The y-axis represents the upstream count. For example, one Desa has 5411 upstream villages

**Fig. 7:**
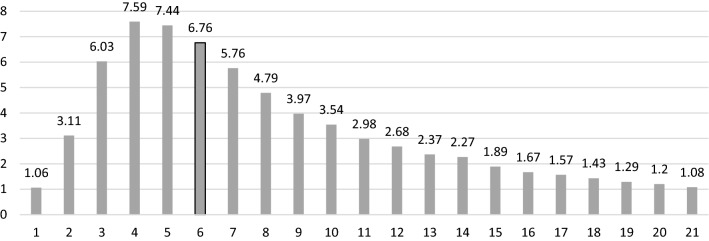
Number of upstream villages > 1%. The x-axis represents the number of villages. The y-axis represents the upstream count. For example, 1.06% of the villages have only one upstream neighbor, and 7.59% of the dataset has 4 upstream villages

The analysis approach taken allows for accurately answering, over large-nation states, the question of which upstream communities contribute to a downstream community across all potential communities. This relationship follows an anticipated exponential pattern as depicted in Fig. 6 with most villages having few upstream villages and a smaller number of downstream villages having a considerable amount of upstream communities. The most common number of neighbors was only 4 (Fig. 7), but one coastal village had as many as 5411 villages upstream of it and its adjacent upstream village one less and so on.

The upstream analysis found that the 13.7 million upstream villages existed for analysis across all years and the average number of upstream villages per village was approximately 55 (Table 1). This is only a small portion of the approximately 6.3 billion combinations of upstream villages theoretically possible if all villages existed on a single linear river network as described above, but this is to be expected in a nation consisting of approximately 17,508 islands which severely limits the maximum upstream villages possible.

**Table 1:** Upstream village summary for all years and all villages including upstream village counts

Year	Unique villages	Number of upstream villages	Average number of upstream villages	Villages > 100 upstream villages	Villages > 250 upstream villages
2008	71,282	3,894,872	55	6729	2982

The full output tables for each village and year can be downloaded from the accompanying Dataverse

We constructed two qualitative and one quantitative method to validate the GIS hydrological process. Firstly, we visually compared the flow accumulations to a digitized river network provided by the World Resource Institute (WRI) to ensure our high flow accumulations and the river data provided by WRI were in close proximity. Although slight deviations of rivers occurred, no major river systems were omitted or had incorrect flow paths. This validation increased confidence in our upstream delineation process.

The digitized rivers layer provided by WRI additionally evidences this facet of our approach. When comparing the upstream village omissions to the river layer, it is apparent in lower quality DEM areas the ArcGIS approach often ceases to count upstream villages even when these villages are upstream and located along rivers that exist the digitized river layer provided.

Secondly, we repeated the entire methodology in ESRI ArcGIS software using the more traditional proprietary version of each of our algorithms. Due to the processing time restraints noted above, we conducted this validation test on a random sample of 14,000 villages. We asked the question; do the upstream villages returned from ArcGIS match those generated by the open-source parallel process selected? ArcGIS consistently returned a lower number of upstream villages per year varying between 23 and 34% lower across the four analysis years. The mismatch rate can also be viewed as a match rate of between 66 and 77%.

Examining why the proprietary and open source upstream village count differs, it appears to be in areas of core SRTM inconsistency, likely caused by canopy interference or SRTM data omissions. In such scenarios, ArcGIS ceases counting upstream villages, even post-hydrologic enforcement, whereas our process continues to accumulate upstream villages. The r.watershed approach used is built explicitly for SRTM data with canopy interference whereas the ArcGIS process is a more general use algorithm not tailored to this situation. ArcGIS appears to fill the DEM in areas of canopy interference to match the canopy artifacts present whereas r.watershed appears to manage such irregularities and continues to work upstream accumulating more villages. The difference is not an actual error, as no one model likely outperforms the other across the varying topography of a nation such as Indonesia. Despite this, the data produced by the r.watershed approach are likely to be more representative than those created by the ArcGIS approach as r.watershed is constructed to account for canopy interference, which is the dominant issue in global SRTM.

Thirdly, we combed our flow accumulation layer, the provided river layer, and the ArcGIS flow accumulation layer with freely available imagery data such as Google Earth, Bing Maps, and the ESRI World Imagery mosaic that showed the rivers did indeed continue upstream in many locations where our data and the ArcGIS data disagreed.

## Conclusion

The approach outlined allows for upstream community behaviors to be modeled accurately across entire basins, nations, or even continents. This allows for downstream health outcomes to be rigorously assessed in the light of upstream activities in a more robust, qualitative, and complete manner. Until now this has only been possible for small isolated basins or in a primarily descriptive manner or in the developed world where drinking from rivers is rare. The need to perform such analysis is primarily required in rural areas of the developing world where data infrastructure is scarce and using rivers for drinking, bathing, and waste disposal are common.

Further enhancement of this process is likely already obtainable. For example, additional post-2017 DEM products such as TanDEM-X that offer global elevation in higher vertical and horizontal resolutions [18] then have been traditionally available to researchers may make this the outlined method even more accurate. Such high-resolution environmental DEMs, the ever-decreasing cost of high-performance computing access, and open-source GIS algorithms that can take advantage of big-data and parallel computing are likely to open a new frontier in the use of GIS in macro health research as it pertains to the role of the environment.

Other algorithmic implementations are possible such as tracing up linear networks, but this does not account for small tributaries and creeks further up the watershed and is also computationally intensive as well as not being as a precise attributing village to the correct river network. Another option not pursued would be to logically assign upstream villages based on logical rules such as assigning a village upstream of a downstream village if it the upstream already been determined to be downstream of a village the object village is downstream of. Such logical rules would likely substantially cut processing time but increase both search and write times. Other algorithmic refinements to speed up the operation are likely plausible as well.

The first significant use of the method and data produced in this paper in health analysis is already published by Garg et al. [8] and use the complete upstream database. They combine the upstream database health survey data to establish that upstream sanitary practices can explain 7.5% of diarrhea-related deaths in Indonesia. Additionally, they find suggestive evidence for differential avoidance behavior in response to different pollutants from downstream communities. The analysis conducted also allowed for additional robustness checks of the database presented such as; Do downstream sanitary behaviors affect upstream health? The negative finding here suggests increased confidence in the database as constructed as upstream health relationships should not be possible.
